# Downregulation of long noncoding RNA B4GALT1-AS1 is associated with breast cancer development

**DOI:** 10.1038/s41598-023-51124-x

**Published:** 2024-02-07

**Authors:** Samaneh ahvaz, Mohammad Amini, Amirhossein Yari, Behzad Baradaran, Asiyeh Jebelli, Ahad Mokhtarzadeh

**Affiliations:** 1 Immunology Research Center, Tabriz University of Medical Sciences, Tabriz, Iran; 2 Department of Biology, Tabriz Branch, Islamic Azad University, Tabriz, Iran; 3 Department of Biological Sciences, Faculty of Basic Sciences, Higher Education Institute of Rab-Rashid, Tabriz, Iran; 4 Clinical Research Development Unit of Tabriz Valiasr Hospital, Tabriz University of Medical Sciences, Tabriz, Iran

**Keywords:** Cancer, Computational biology and bioinformatics, Genetics, Molecular biology

## Abstract

The misregulation of long non-coding RNAs (lncRNAs) is related to the progressive evolution of various human cancers, such as Breast cancer (BC). The role of lncRNA B4GALT1-AS1 has been investigated in some human cancers. Therefore, studying B4GALT1-AS1 expression was aimed for the first time in the tumor and marginal tissues of BC in this study. The cancer genome atlas (TCGA) database was utilized to evaluate the relative expression of B4GALT1-AS1 in BC and other cancers. RNA was extracted from twenty-eight paired BC and marginal tissues, and cDNA was synthesized. The quantitative expression level of B4GALT1-AS1 was evaluated using real-time PCR. The bioinformatics analyses were performed to identify co-expression genes and related pathways. B4GALT1-AS1 was significantly downregulated in BC specimens compared to tumor marginal samples. The TCGA data analysis confirmed the downregulation of B4GALT1-AS1 in BC. The bioinformatics analysis discovered the correlation between 700 genes and B4GALT1-AS1 and identified GNAI1 as the high degree gene which was positively correlated with B4GALT1-AS1 expression. It seems B4GALT1-AS1 provides its function, at least partly, in association with one of the hippo pathway components, YAP, in other cancers. This protein has the opposite role in BC and its loss of function can result in poor survival in BC. Further research is needed to investigate the interaction between B4GALT1‐AS1 and YAP in various subtypes of BC.

## Introduction

Breast cancer (BC) is known as the second most common cancer with two million new cases in women reported in 2020. BC incidence has been increased over the past three decades^1,2^. Patients with BC can be diagnosed in the early stages. Consequently, further studies are needed to demonstrate new molecular goals for better management of BC. Many factors such as epigenetic, genetic, and environmental have been recognized to be involved in BC progression^3^.

long non-coding RNAs (lncRNAs) are one of the important epigenetic factors^3^. They are a small collection of RNA transcripts with more than 200 nucleotides in length that have an important role in the regulation of gene expression^4–6^. lncRNAs have been illustrated to participate in numerous biological processes, such as cell proliferation, cell differentiation, apoptosis, invasion, and migration^7,8^. Studies indicate that lncRNAs participate in metastasis, tumorigenesis, and progression of various cancers including BC. While some lncRNAs can act as tumor-enhancing agents, others provide a role as tumor inhibitors^9–11^. Some lncRNAs, such as MALAT1 and HOTAIR, are expressed through breast tumorigenesis, showing oncogenic properties; whereas others like GAS5 and XIST function as tumor suppressors^12^.

B4GALT1 Antisense RNA 1 (B4GALT1-AS1) belongs to the lncRNA class. The chromosomal position of lncRNA B4GALT1-AS1 is at 9p21.1. This lncRNA sequence has 508 nucleotides length and is found in Homo sapiens^13^. Studies have highlighted the importance of B4GALT1-AS1 in the formation or progression of cancers such as colorectal cancer (CRC), Non-small cell lung cancer (NSCLC), and osteosarcoma cancer (OS). B4GALT1- AS1 upregulated in CRC, OS, and NSCLC cells functions as an oncogene^14–16^. However, no study has been performed to evaluate the expression of B4GALT1-AS1 in BC. Therefore, this research aimed to inspect the correlation between B4GALT1-AS1 expression and tumorigenesis of BC. In this regard, we initially evaluated the expression level of B4GALT1-AS1 in BC samples with an analysis of the cancer genome atlas (TCGA). Subsequently, the expression of B4GALT1-AS1 was assessed in the tumor and marginal tissue samples of BC patients using the real-time PCR method. Moreover, the relationship between the expression level of B4GALT1-AS1 and clinical pathological parameters was analyzed.

## Methods and materials

### Datasets and data processing

Breast cancer genome atlas (TCGA-BRCA) RNA-seq gene expression data was obtained using the TCGAbiolinks package^17^, then normalized and processed with the limma package^18^ in the R programming language. Using GraphPad Prism, we were able to examine the ROC curve and expression level of the B4GALT1-AS1 non-coding gene. The expression of this lncRNA was analyzed across many cancer types using the Ualcan database^19,20^. To investigate the correlation between the expression of B4GALT1-AS1 and the pathogenic data, the TCGA RNA seq was queried using the Ualcan online tool. Other datasets were evaluated to further assess the expression level of B4GALT1-AS1. Gene Expression Omnibus (GEO) accession codes GSE156229 and GSE139274 are unique to these two datasets that were collected from the GEO database.

### Preparation of Sample

Twenty-eight BC and 28 paired tumor marginal tissue samples were obtained from Imam Reza Hospital of Tabriz from 2019 to 2020. Written informed consent was obtained from all patients. All patients underwent no chemotherapy or radiation therapy before surgery. Samples were frozen in liquid nitrogen and stored at − 80 °C until RNA extraction. All clinicopathological features including tumor size, age, site of primary tumor, lymphatic and vascular invasion, serosal invasion, histological grade, clinical stage and GC family history were presented in patient profiles.

### RNA extraction and cDNA synthesis

Tissue samples were ground in liquid nitrogen using a mortar and pestle, then transferred to a lysis buffer and homogenized with a needle and syringe. Total RNA was extracted with Trizol reagent (Brand Riboex manufactured by Gene All) according to the protocol instructions. The quantity and quality of RNA were calculated with a NanoDrop spectrophotometer and electrophoresis on 1% agarose gel, respectively. Then, cDNA was synthesized using the PrimeScriptTM RT Reagent Kit (Takara Bio, Japan) from 1 μg of total RNA in a final volume of 20 µl according to the standard procedure (50 °C in 30 min for reverse transcription, 95 °C in 5 min for inactivation of RT enzyme, 10 °C in infinitive for end) by the thermocycler PCR.

### Real-time PCR (qPCR)

Real-Time PCR was performed using the BioFACT™ 2X Real-Time PCR Master Mix and gene-specific primers. The total reaction volume was set in a 10 μl. The reaction was performed in three steps as follows: Step 1: 95 °C in 13 min for holding stage or initial heat. Step 2: 45 cycles for denaturation at 95 °C in 10 s, primer annealing at 60 °C in 30 s, and extension at 72 °C in 20 s. Step 3: at the end of each run melting curves were obtained. The *GAPDH* gene was used for data normalization. The expression of target genes was calculated using the 2^-ΔΔCt^ method. NCBI Primer-3 online website, (https://www.ncbi.nlm.nih.gov/tools/primer-blast/) was used to design primers. In Table 1 qPCR primer sequences are provided.

**Table 1:** qPCR primer sequences.

Target gene	Forward (5′–3′)	Reverse (5′–3′)
B4GALT1-AS1	GCATCAGAGAGAATATGGAAGG	GGCTTAATAGTTGGTTCAGTG
GAPDH	AAGGTGAAGGTCGGAGTCAAC	GGGGTCATTGATGGCAACAA

### Functional enrichment analysis

To identify B4GALT1-AS1-related pathways, we isolated the B4GALT1-AS1 co-expression genes using the web resources lncHUB and GEPIA. This group of genes was then subjected to a pathway enrichment study. Pathway enrichment analysis was done using the web-based tool Enrichr to query the Kyoto Encyclopedia of Genes and Genomes (KEGG) database to identify biological pathways impacted by these genes. In the end, the data was represented graphically in the Cytoscape software.

### Statistical analysis

The analysis of qPCR results and drawing the graphs were performed using GraphPad Prism 6. Differences in *B4GALT1-AS1* expression level between samples were determined using the Wilcoxon matched-pairs signed rank test, paired and unpaired t-test, and Mann–Whitney test. A significant P value was measured to less than 0.05.

### Ethics approval

The ethical committee of the Immunology Research Center, Tabriz University of Medical Sciences approved the study. Written informed consent was obtained from all patients**.**

## Results

### B4GALT1-AS1 was considerably downregulated in BC

**Figure 1:**
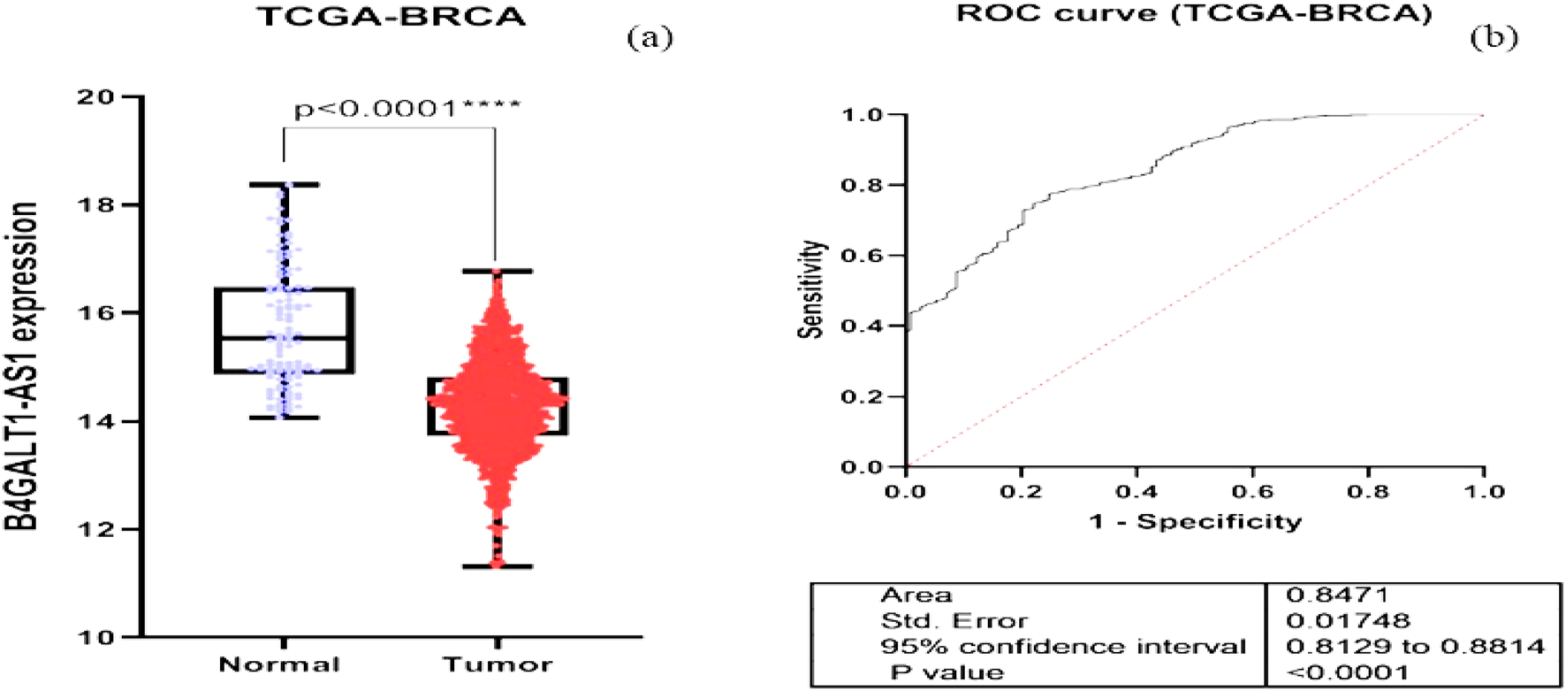
(**a**) The expression level of B4GALT1-AS1 in the TCGA-BRCA dataset. The expression level of B4GALT1-AS1 was significantly reduced in breast cancer samples compared to normal tissues (**b**) The correlation between the Rock curve and TCGA-BRCA level to differentiate between patients and normal individuals. An area under the ROC curve (AUC) of 0.8 to 0.9 is considered excellent discrimination, suggesting B4GALT1-AS1 may be useful as a diagnostic biomarker.

**Figure 2:**
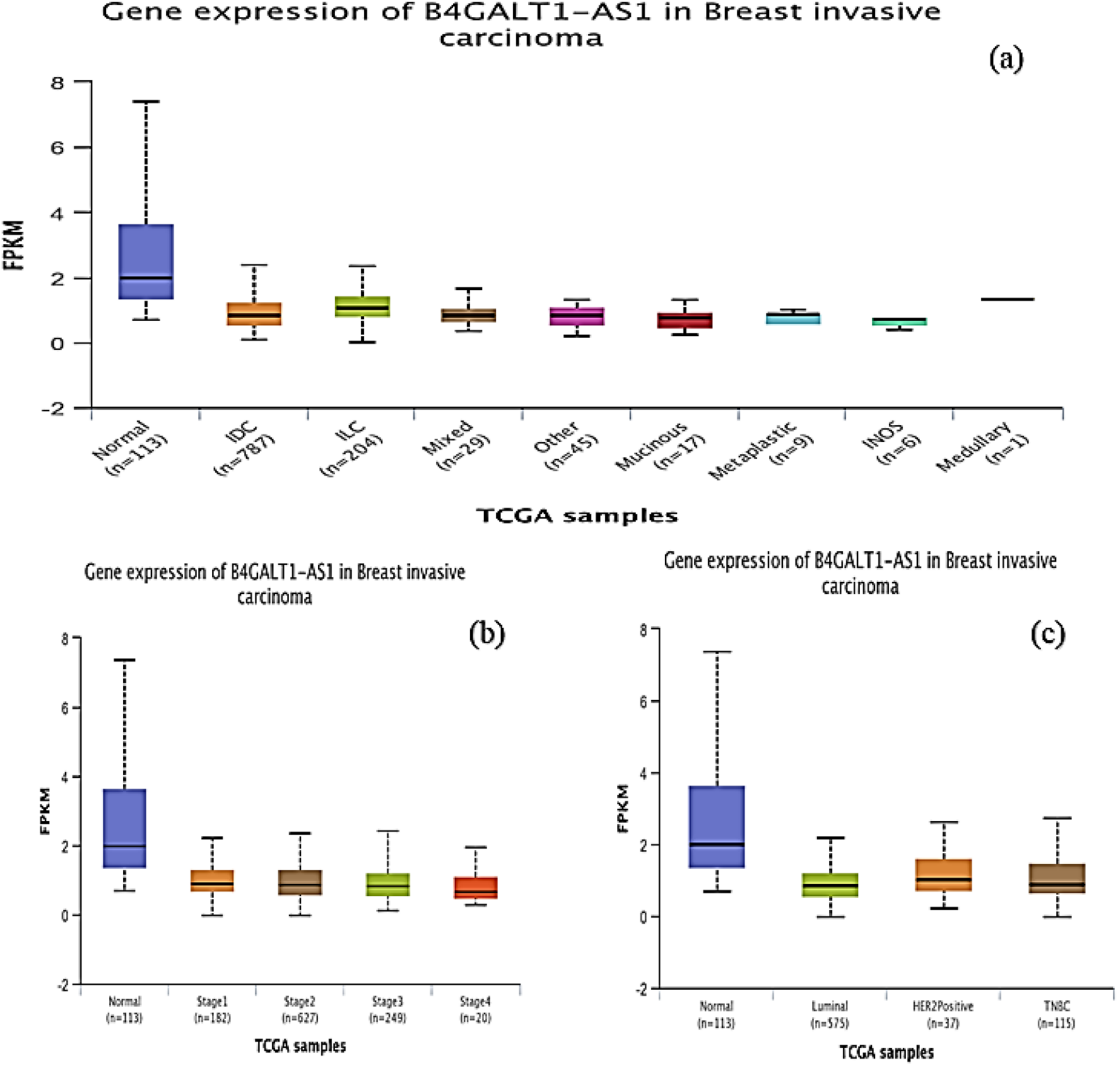
B4GALT1-AS1 expression and its correlation with tumor histology (**a**), Stages (**b**) and major subclasses (**c**). The significant reduced expression of B4GALT1-AS1 in all stages and different subtypes of breast cancer introduces its possible role in breast cancer progression.

**Figure 3:**
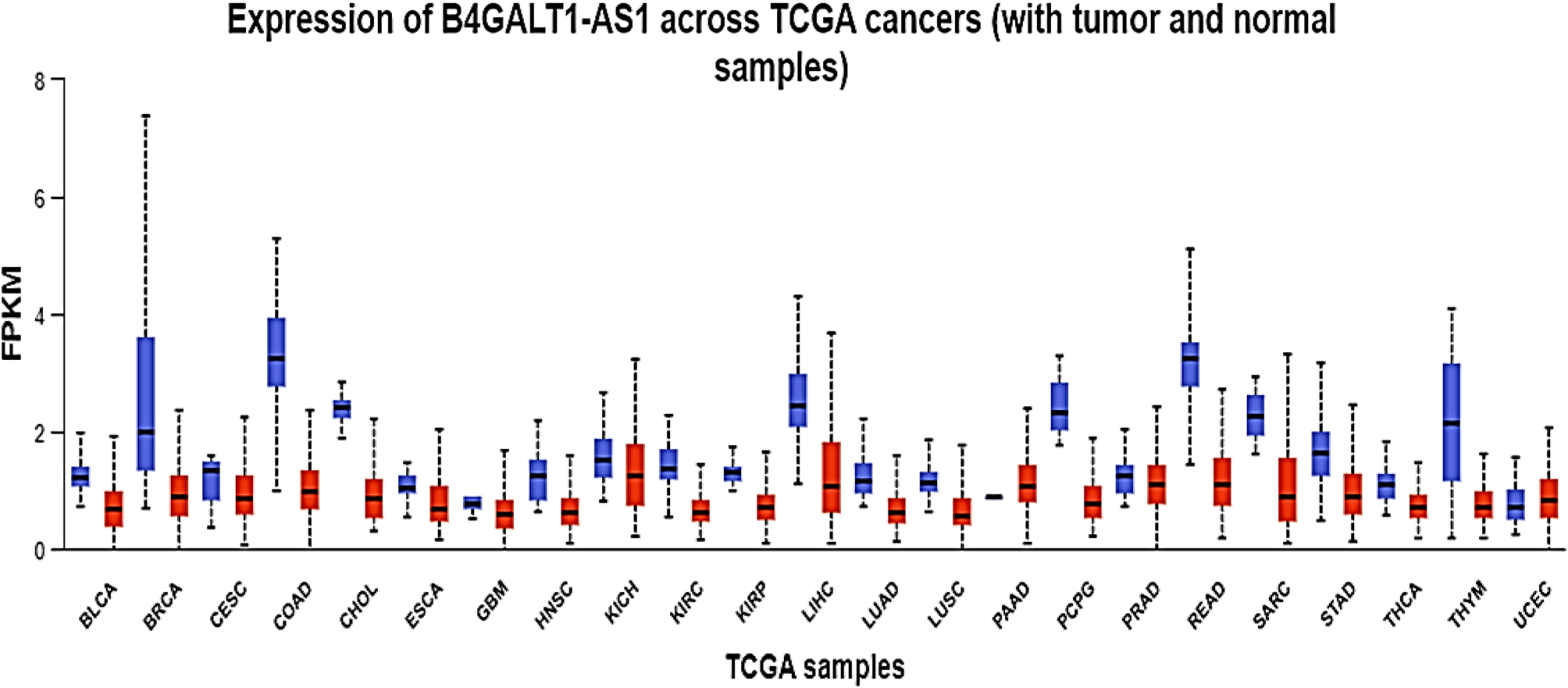
Downregulation of B4GALT1-AS1 was shown in several cancers, most notably breast cancer. This graph demonstrates the significance of this lncRNA in tumorigenesis of many different cancer types.

**Figure 4:**
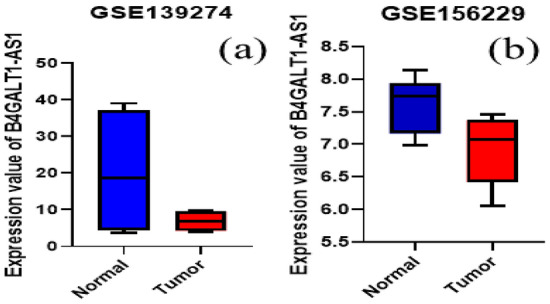
The expression level of B4GALT1-AS1 in GSE139274 (**a**) and GSE156229 (**b**) dataset. The results of both datasets was in accordance with TCGA-obtained data.

The data on BC's transcriptome obtained from TCGA demonstrated that B4GALT1-AS1 was considerably downregulated in the disease. Analysis of RNA-seq datasets from the TCGA showed that the expression level of B4GALT1-AS1 was decreased significantly in breast cancer compared to normal tissues (Fig. 1a). The potential of this lncRNA to serve as a diagnostic biomarker was then assessed. The receiver operating characteristic curve (ROC) and Area Under Curve (AUC) were plotted on a GraphPad prism software to demonstrate the accuracy of the data collection process (Fig. 1b). The results of the study demonstrated that the B4GALT1-AS1 expression level was drastically reduced in various breast cancer kinds and stages (Fig. 2). According to the pan-cancer investigation of the Ualcan database, the expression level of B4GALT1-AS1 was downregulated in different kinds of cancer and particularly in breast cancer (Fig. 3). Besides, the expression level of B4GALT1-AS1 lncRNA was assessed using other datasets. These two Gene Expression Omnibus datasets have unique GEO accession codes: GSE156229 and GSE139274 (Fig. 4). The datasets utilized in this investigation are detailed in Table 2.

**Table 2:** The dataset characteristics in detail.

Datasets	Normal samples	Breast cancer samples	Platform
TCGA-BRCA	114	1095	RNA sequencing
GSE156229	6	6	GPL22120: Agilent-078298 human ceRNA array V1.0 4X180K
GSE139274	4	4	GPL20795: HiSeq X Ten (Homo sapiens)

### B4GALT1-AS1 was downregulated in BC samples

**Figure 5:**
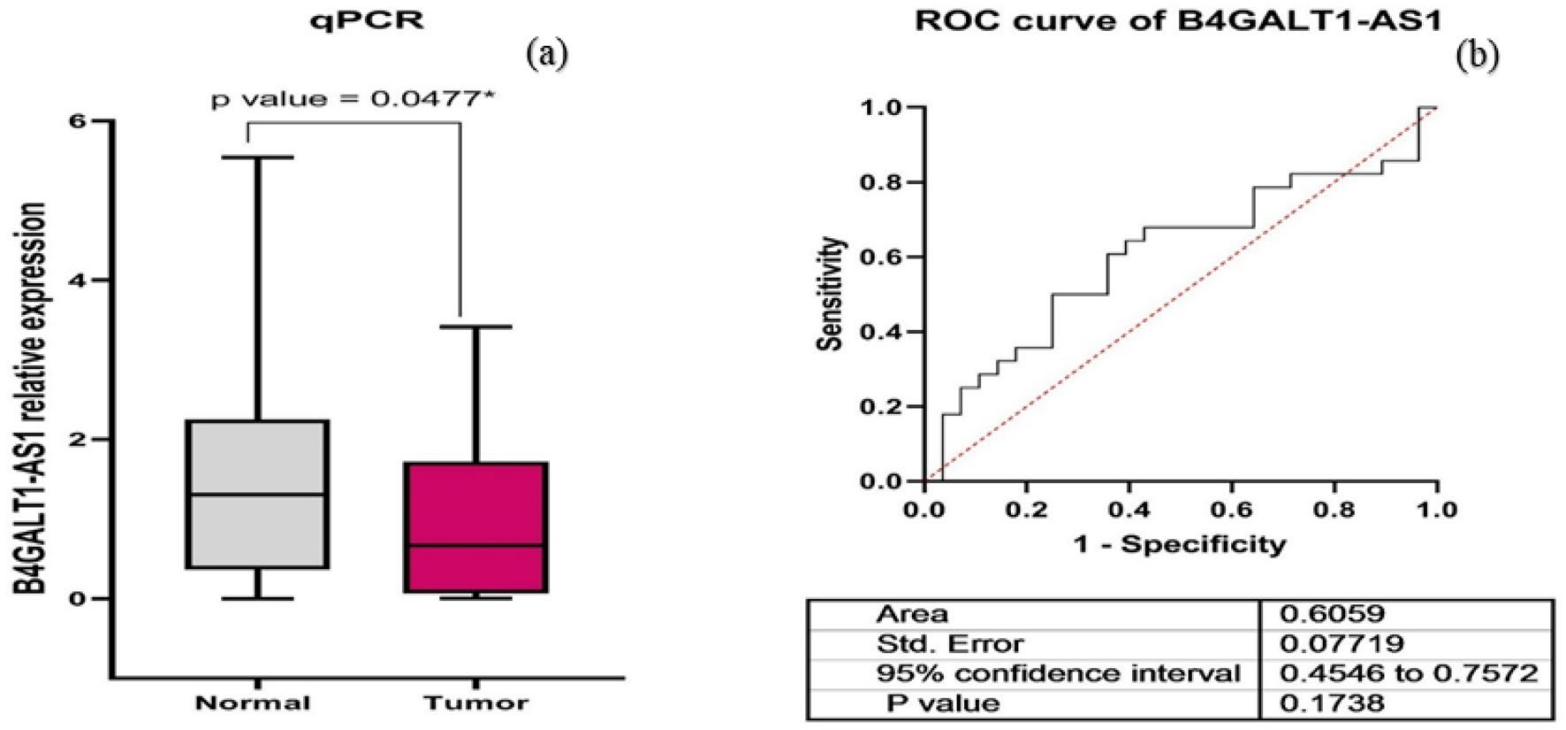
(**a**) The analysis of B4GALT1-AS1 expression in tumor (N = 28) and marginal tissues (N = 28) using the qPCR technique. Similar to TCGA and GEO data, qPCR results showed a significant reduction in the expression of the B4GALT1-AS1 lncRNA (**b**) Rock curve for B4GALT1-AS1 to differentiate tumor tissues from marginal samples.

The expression of B4GALT1-AS1 was investigated in 28 BC tumor and 28 margin samples using qPCR. The analysis of qPCR data revealed that the expression of B4GALT1-AS1 was significantly reduced (*p*-value = 0.0477) in BC tissue samples compared to paired tumor margin samples (Fig. 5a), which confirmed the results of TCGA and GEO datasets. Rock curve analysis indicated that the expression of B4GALT1-AS1 was not significant in differentiating tumor tissues from marginal samples (Fig. 5b). However, in TCGA samples which included a large volume of patients, it could be significant and specifically differentiate between patients and normal individuals.

### Pathological examination of samples

**Figure 6:**
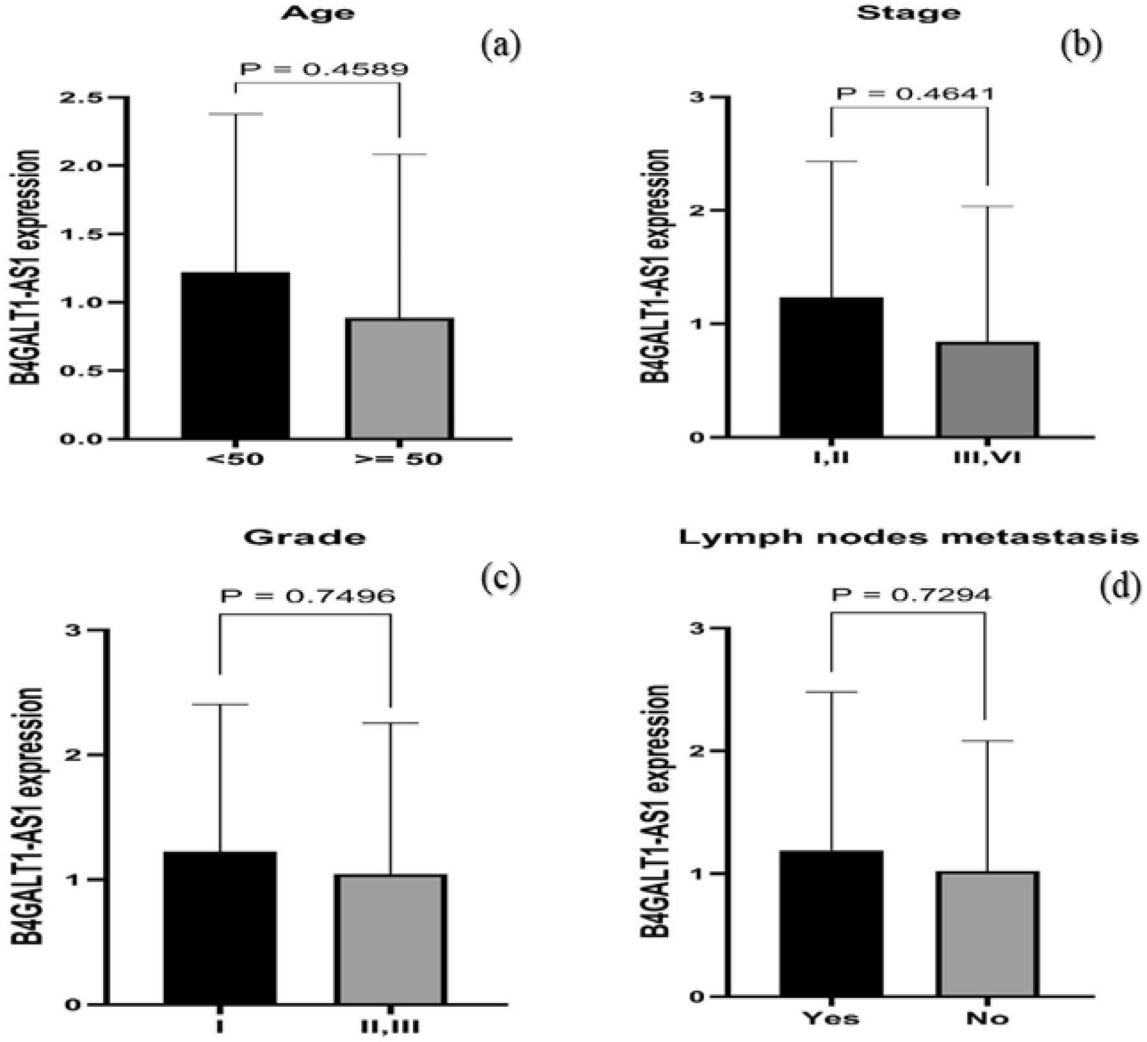
The relationship between B4GALT1-AS1 expression and pathological features in BC patients. (**a**) Age (*p* = 0.4589), (**b**) stage (*p* = 0.4641), (**c**) grade (*p* = 0.7496), (**d**) Lymph nodes metastasis (*p* = 0.7294). The un-paired t-test was performed for the statistical analysis.

The relationship between pathological features and *B4GALT1-AS1* expression level was analyzed in BC patients. There was no significant association between *B4GALT1-AS1* expression and age (*p* = 0.4589) (Fig. 6a), clinical stage (*p* = 0.4641) (Fig. 6b), histological grade (*p* = 0.7496) (Fig. 6c), and lymph nodes metastasis (*p* = 0.7294) (Fig. 6d). Interestingly, five of the patients with the highest levels of B4GALT1-AS1 were 46 years or younger, which may be correlated with early BC.

### B4GALT1-AS1 is vital in a broad number of biological functions

**Figure 7:**
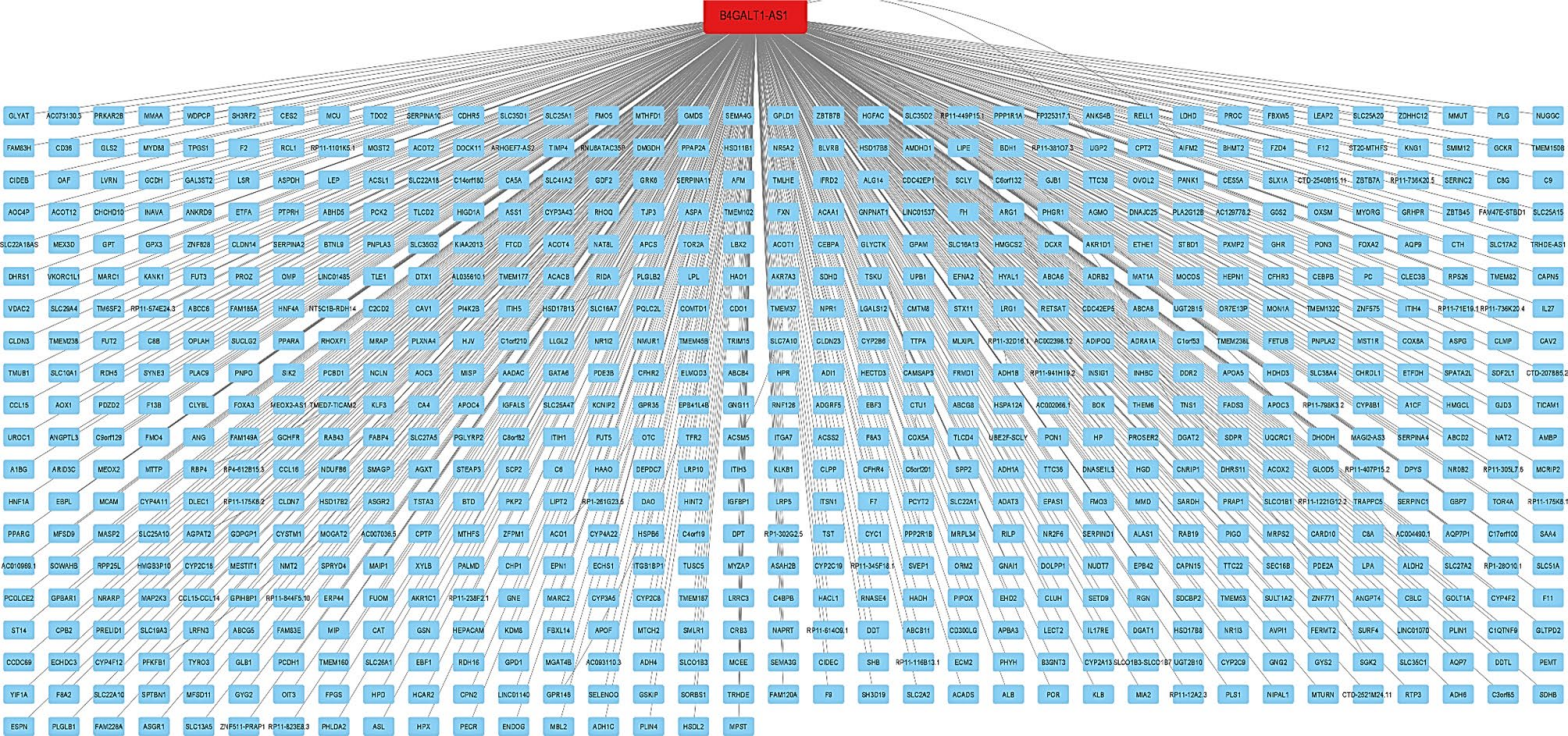
The network of genes that are expressed together. Nodes in blue represent mRNAs, whereas node in red represents B4GALT1-AS1.

**Figure 8:**
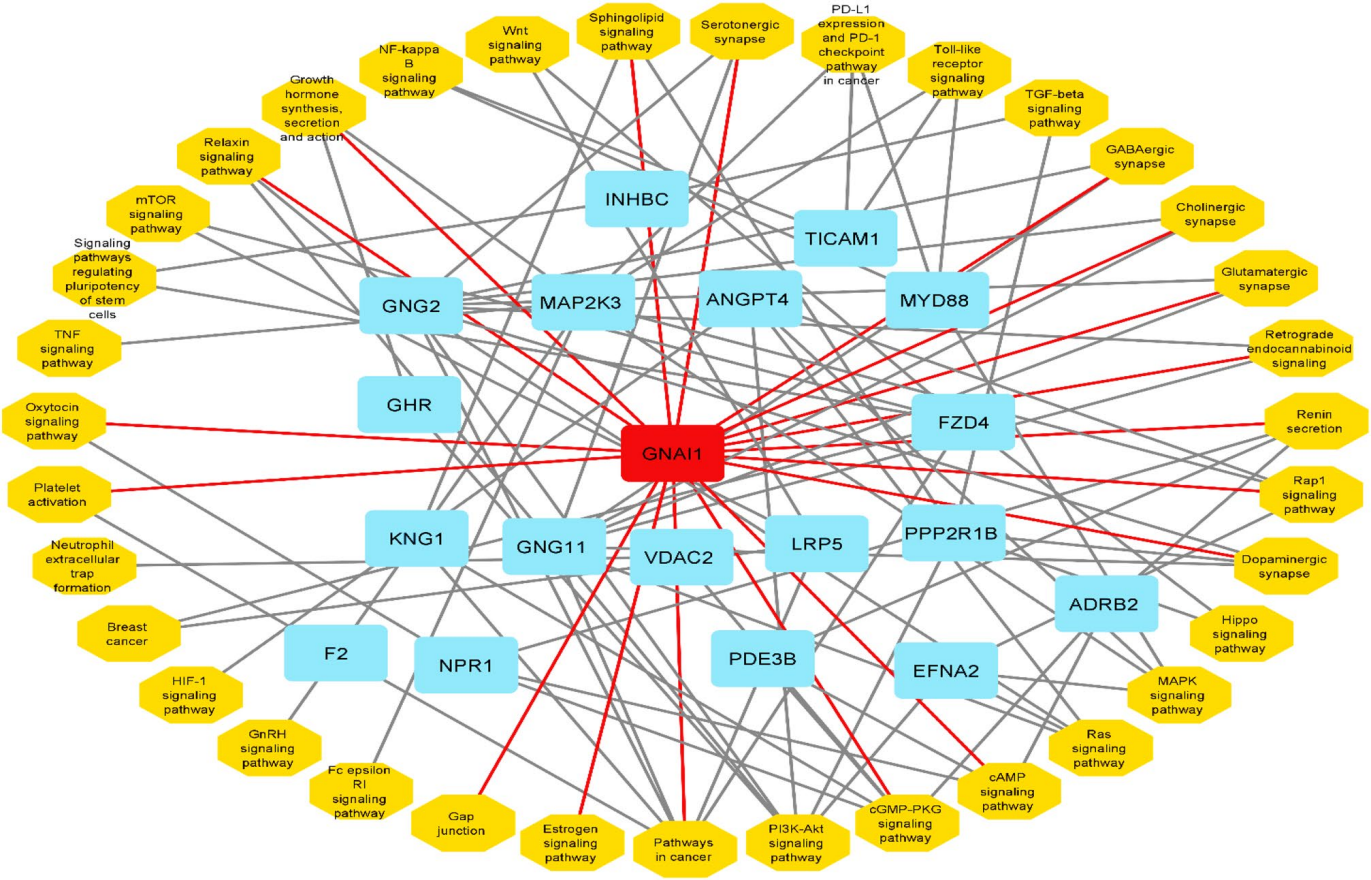
Related pathways and genes that are targets of B4GALT1-AS1. They function as a big network. In this network, genes and pathways are shown in blue and orange nodes, respectively. Red node shows the high degree gene in association with B4GALT1-AS1.

**Figure 9:**
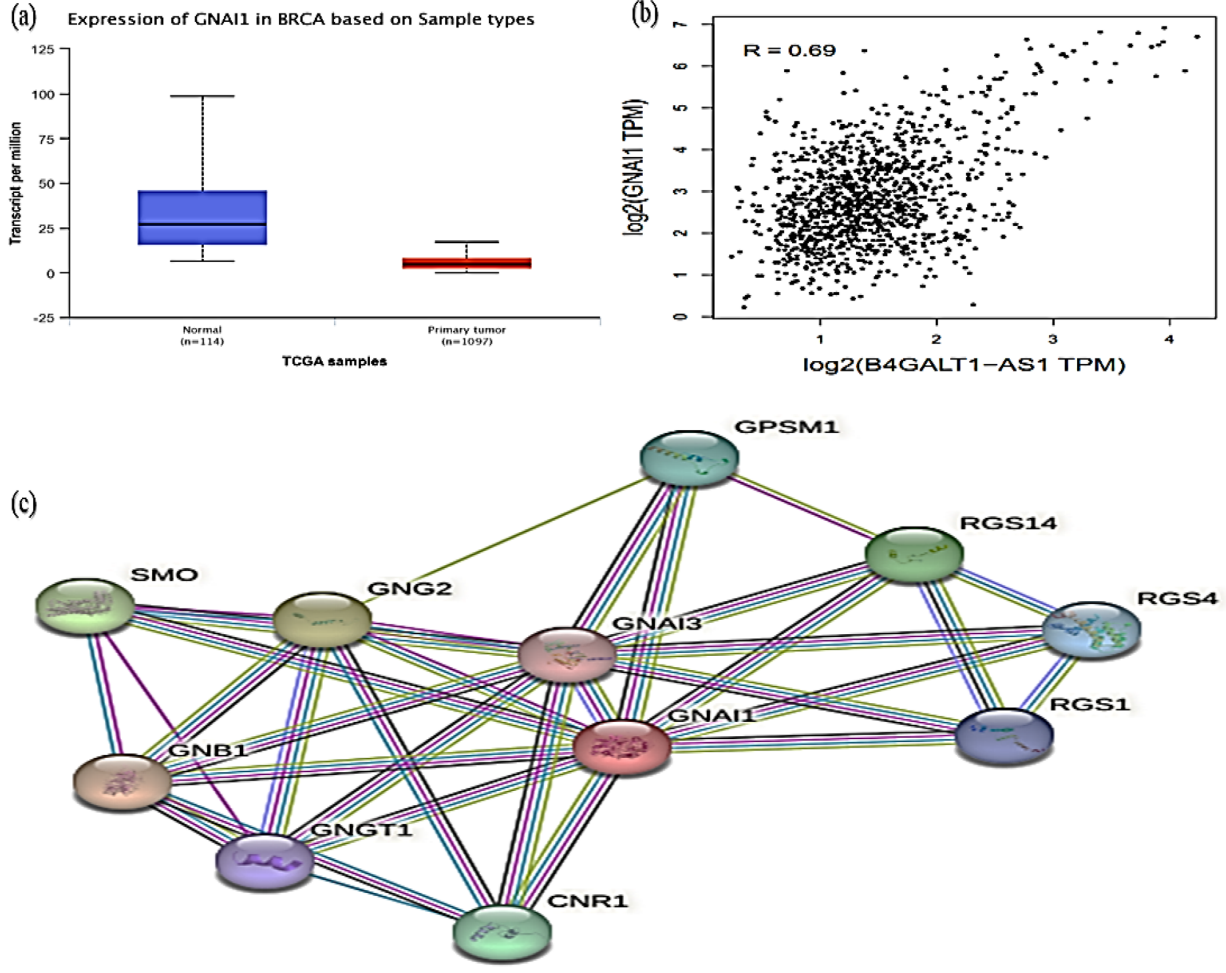
(**a**) The relative expression of GNAI1, (**b**) Pearson correlation and (**c**) protein interactions related to GNAI1. Similar to B4GALT1-AS1, GNAI1 was downregulated in breast cancer, representing a positive correlation between B4GALT1-AS1 and GNAI1.

**Figure 10:**
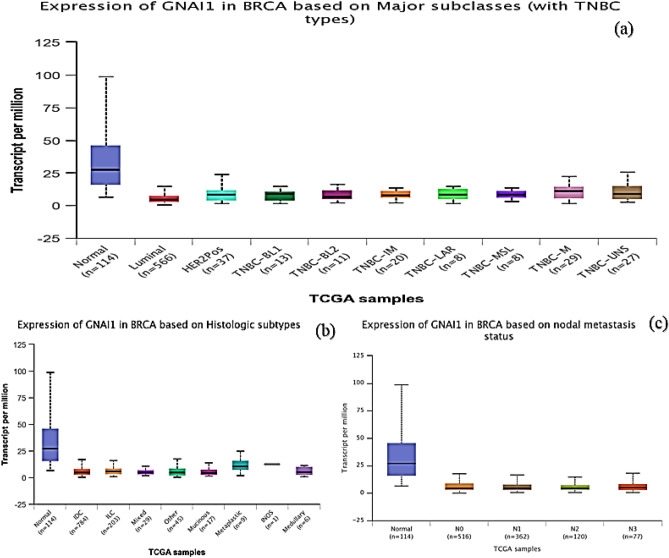
GNAI1 expression and its correlation with major subclasses (**a**), tumor histology (**b**), and nodal metastasis (**c**). The results shared similarity with findings observed for B4GALT1-AS1. The remarkable reduced expression of B4GALT1-AS1 and GNAI1 in all stages and different subtypes of breast cancer emphasize a positive correlation between these two genes.

To identify co-expressed genes associated with B4GALT1-AS1, we searched the TCGA database with the use of the online tools lncHUB (https://maayanlab.cloud/lnchub/) and gepia (http://gepia.cancer-pku.cn/). Thus, 700 genes were found to have expression levels that are correlated with B4GALT1-AS1. Figure 7 depicts the co-expression network. The Enrichr online tool (https://maayanlab.cloud/Enrichr/) was then used to query the KEGG pathway database, demonstrating the genes' important involvement in the various pathways once they were imported. Figure 8 displays the pathways associated with the co-expressed genes. The G Protein Subunit Alpha I1 (GNAI1) gene was identified as the high-degree gene in the pathway network. Figure 9 displays the expression level, Pearson correlation analysis, and protein interaction network of the *GNAI1* gene. According to data retrieved from the Ualcan TCGA base online tool, GNAI1 was profoundly downregulated in breast cancer. Furthermore, data obtained from the Gepia online tool's Pearson correlation analysis showed that B4GALT1-AS1 and GNAI1 were positively and significantly correlated in breast cancer. Finally, retrieved information from the String database revealed GNAI1’s protein connections (Fig. 9). The investigation found that GNAI1 expression was significantly lower across all subtypes and stages of breast cancer (Fig. 10). Figure 7.

## Discussion

BC is the most famous cancer among women and the leading cause of death in the world^24^. BC is diagnosed using physical examinations and special techniques such as mammography^25^. Early detection of benign and malignant tumors in the early stages and proper treatment is difficult^26^. Therefore, the identification of molecular markers for the diagnosis of this cancer in the early stages is of particular importance^27^.

Many studies have shown that lncRNAs have an important function in the biology of cancers and are out of regulation in various cancers^11,28^. It has been shown that the expression of certain lncRNAs is involved in the development and progression of BC, highlighting the different expression patterns of lncRNAs in BC tissues compared to normal breast samples^12,29^. Due to the central task of lncRNAs in the development or progression of cancers and tumorigenesis, in the present study, a lncRNA called B4GALT1-AS1 was selected and its role in BC was evaluated. To date, no study has been performed to evaluate the association of B4GALT1-AS1 expression with BC.

This research aims to better understand the function of the noncoding RNA B4GALT1-AS1 in the development and progress of breast cancer. For this purpose,, after pathological examination of the samples, RNA extraction and cDNA synthesis were performed on all tumor and marginal tissue samples. Then real-time PCR reaction was performed for gene expression. Analysis of the results showed that the expression of B4GALT1-AS1 in BC tumor tissue was significantly reduced compared to marginal tissues. Besides, no significant association was observed between clinical pathological parameters (age, stage, grade, and lymph node metastasis) and the decreased expression of B4GALT1-AS1. This was probably due to the small number of samples in this project, so the pathological parameters should be examined in a large number of samples and a wide geographical area to obtain precise results.

The expression of B4GALT1-AS1 in other cancers including OS, CRC, and NSCLC tissues was evaluated in previous studies, and it has been found that B4GALT1-AS1 was upregulated in the mentioned cancers^16,30^. In NSCLC, the proliferative capacity of A549 and H1299 cells was reduced by B4GALT1-AS1 knockdown. The evidence showed that B4GALT1-AS1 by increasing the isolation of miR-30e resulted in increased expression of transcription factor (SOX9)^16^. Furthermore, the interaction between B4GALT1-AS1 and miR-144-3p regulated ZEB1 expression and increased the progression and tumorigenesis of the NSCLC cell line^31^. Further studies highlighted that the function of B4GALT1-AS1 was associated, at least partly, with two key proteins, HuR and YAP, which have been identified to provide roles in the development of cancers^32^. YAP-associated protein (YAP) is a prolin-rich phosphoprotein encoded by the *yap1* gene^33^ and is a member of the hippo pathway. This pathway participates in the regulation of cell proliferation, survival, and apoptosis; hence plays an important role in the control of organ development^34^. HuR is a ubiquitously expressed RNA-binding protein that binds and stabilizes AU-rich element-containing mRNAs that encode proto-oncogenes, growth factors, and cell cycle regulators in several cancer types^35^.

Li et al.^15^ reported a significant increase in the expression of B4GALT1-AS1 in OS. B4GALT1-AS1 is directly bound to HuR to boost the stability of YAP mRNA and thus increase its transcription activity. In cells with B4GALT1-AS1-knockdown, HuR nuclear-cytoplasmic translocation was inhibited and YAP mRNA stability/expression was reduced. All findings suggest the function of B4GALT1-AS1 as a co-activator of HuR on the progression of OS cells; however, it is uncertain whether this phenomenon is common, which requires more exploring in the future^36^.

The upregulation of B4GALT1-AS1 was also reported in CRC cells. The direct binding of B4GALT1-AS1 to YAP regulated CRC cell growth and increased YAP transcriptional activity. Although the YAP/TAZ mRNA level was not changed following B4GALT1‐AS1 knockdown, the protein level of YAP (but not TAZ) was remarkably reduced by B4GALT1‐AS1 knockdown. These results proposed that B4GALT1‐AS1 functioned as a YAP coactivator in promoting CRC cell stemness. However, the underlying mechanisms related to the effect of B4GALT1‐AS1 knockdown on the reduction of YAP protein level are still unclear. A possible reason can be considered as the effect of B4GALT1‐AS1 knockdown on facilitation of YAP translocation from nucleus to cytoplasm which is responsible for ubiquitination-mediated YAP degradation^30^.

These two studies highlighted the critical relationship between B4GALT1‐AS1 and YAP protein. While YAP overexpression results in tumor progression and worse survival in particular malignancies, YAP can function as a tumor suppressor and induce apoptosis. Loss of function of YAP has only been observed in BC^37^. YAP knockdown in BC cell lines increased the invasion, migration, and metastasis ability^38,39^. YAP is located on the position of q22-23 of chromosome 11, a locus in which repetitious loss of heterozygosity (LOH) occurs in BC. Loss of LOH in this position has been related to poor survival in BC, suggesting that it plays a tumor suppressor role in BC oncogenesis^40,41^. The compelling evidence indicates there might be a different regulatory mechanism associated with YAP on BC in comparison to other solid tumors. It can, at least partly, explain the different expression of B4GALT1‐AS1 reported in BC compared to other cancers. Further research is needed to investigate the interaction between B4GALT1‐AS1 and YAP in various subtypes of BC.

To further investigate B4GALT1‐AS1 related pathways and target genes, a combination of bioinformatics and systems biology techniques was used in this research. This was accomplished by isolating the genes expressed with the target lncRNA of interest, and then using computational tools to explore the corporates' pathways. After investigating the data and the connections between nodes, we decided to go more into one of the co-expressed genes, GNAI1, which is involved in a wide variety of biological processes.

Importantly, bioinformatics analyses discovered a positive correlation between B4GALT1-AS1 and GNAI1 and also predicted downregulation of GNAI1 in BC. GNAI1, encoding the subunit α of guanine nucleotide-binding protein, is a member of Gα inhibitory family and extremely expressed in immune cells. It takes part in G protein-coupled receptor (GPCR) and also non-GPCR signaling pathways^42^. GNAI1 transducing extracellular signals functions in several physiologic processes including proliferation and differentiation^43,44^. The high GNAI1 expression phenotype was reported in HR(+)/HER2(−) BC in adolescents and young adults which was correlated to ether lipid metabolism and complement and coagulation cascades. These phenomena promote tumor growth by affecting gene expression and regulating the inflammatory response, epithelial-mesenchymal transition and angiogenesis. These findings introduce GNAI1 as a likely therapeutic target for HR(+)/HER2(−) BC^45^. However, further studies are necessary to research the function of GNAI1 in other BC subtypes and correlation with B4GALT1-AS1.

The overall results suggest that the mechanisms of tumor progression are possibly different in breast tissue from other tissues and may depend on the type of BC cells. The mechanisms associated with HuR and YAP appear to vary according to the type of mutations and genes involved in BC. However, more large-scale studies with larger samples are needed to achieve a more accurate and complete result.

## Conclusion

Despite extensive research, BC remains the leading cause of cancer death in women. Therefore, further studies are needed to investigate the molecular mechanisms involved in BC progression and metastasis. These studies may help establish new biomarkers or targets for the prognosis and treatment of the disease. Emerging evidence has shown that lncRNAs may act as oncogenes or tumor suppressors and therefore introduce them as potential biomarkers or therapeutic targets for cancer patients. In the present study, the expression of B4GALT1-AS1 in BC was investigated. Significant changes in its expression were observed in tumor tissues compared to marginal ones. It seems the expression pattern of B4GALT1-AS1 is different in BC tissues in comparison to other tissues from OS, CRC, and NSCLC, highlighting a possible different regulatory mechanism related to this gene. Regulatory mechanisms focusing on YAP and GNAI1 may provide useful research aspects for further studies.

## Data Availability

Data are openly available in this article.
